# A phase retrieval algorithm for triply periodic minimal surface like structures

**DOI:** 10.1107/S2053273322010786

**Published:** 2023-01-01

**Authors:** Toshihiko Oka

**Affiliations:** a Department of Physics, Faculty of Science, Nanomaterials Research Division, Research Institute of Electronics, Shizuoka University, Shizuoka, 422-8529, Japan; University of Patras, Greece

**Keywords:** crystallographic phase retrieval, lyotropic liquid crystals, mesoporous silica, triply periodic minimal surfaces

## Abstract

A method to solve the crystallographic phase problem of materials with triply periodic minimal surface like structures, such as lyotropic liquid crystal bicontinuous cubic phases, is reported. In triply periodic minimal surface like structures, the difference between the maximum and minimum electron densities tends to be the smallest for the true phase combination among the possible combinations [Oka (2022). *Acta Cryst.* A**78**, 430–436]. Using this feature, a new iterative phase retrieval algorithm for structure determination was developed. The algorithm modifies electron densities outside the upper and lower thresholds in the iterative Fourier transformation process with fixed amplitudes for the structure factors, and efficiently searches for the structure with the smallest difference between the maximum and minimum electron densities. The proper structure was determined by this algorithm for all tested data for lyotropic liquid crystal bicontinuous cubic phases and mesoporous silicas. Although some cases required constraints such as the volume fraction for structure determination, more than half could be determined without any constraints, including space groups.

## Abstract 2

A method to solve the crystallographic phase problem of materials with triply periodic minimal surface like structures, such as lyotropic liquid crystal bicontinuous cubic phases, is reported.

## 1. Introduction

Crystallography serves as a fundamental method to offer structural information for the understanding of materials. This statement applies to both typical, *i.e.* well ordered, crystals, and highly disordered systems such as liquid crystals. However, the application of crystallographic methods to the latter is very difficult because of the limited number of reflections available. The bicontinuous cubic phase of lyotropic liquid crystals (LLC) has a triply periodic minimal surface (TPMS) like structure and three-dimensional periodicity with natural beauty (Hyde *et al.*, 1996 ▸). Thus, these systems are good targets for structural studies. The structure of the LLC bicontinuous cubic phase was first determined by the pioneering work of Luzzati *et al.* using X-ray powder diffraction (Luzzati *et al.*, 1988 ▸; Mariani *et al.*, 1988 ▸). Recently, the present author established a single-crystallization method for the LLC bicontinuous cubic phase (Oka & Hojo, 2014 ▸), and performed structural analyses of the LLC bicontinuous cubic phases while considering model structures (Oka, 2017 ▸; Oka *et al.*, 2018 ▸, 2020 ▸). The essential difficulty is the so-called phase problem, which is still unsolved.

Numerous researchers have tackled the phase problem (Sayre, 2015 ▸). Direct methods and variants are routinely used for structure determination: it is impossible to imagine the current practice of crystallography without them (Giacovazzo, 2001 ▸). These methods are based on the general properties of crystals. The charge-flipping method, which has a simple iterative algorithm for structural determination, utilizes the fact that the positive electron density of atoms is concentrated in a small region, while the remaining regions have zero electron density (Oszlányi & Sütő, 2004 ▸, 2008 ▸; Palatinus, 2013 ▸). Its success implies the possibility of using the structural features of highly disordered systems if we can identify suitable expressions for the features.

In a previous paper (Oka, 2022 ▸), the author proposed two indicators reflecting the plausibility of phase combinations of experimental data for the LLC bicontinuous cubic phase. The indicators are based on the structural features of materials: the electron density tends to be constant in the direction in which liquid crystal molecules diffuse. This property suggests that the continuity of the density is a good indicator. The difference density between the maximum and minimum (*I*_ρ_) seems to be a good and simple indicator. Another indicator (*I_K_*), which utilizes the Hessian matrix of the electron density, is also acceptable. In the previous paper, the electron density and indicators were calculated for all possible phase combinations for the test data with centrosymmetric space groups. The result showed that the two indicators work well. Although the potential utility of the method based on the two indicators was confirmed for LLC bicontinuous structures, testing all phase combinations becomes impractical with an increase in the number of independent reflections. In addition, the method is only applicable to centrosymmetric space groups.

In this paper, an iterative phase retrieval algorithm for structure determination to overcome these difficulties is proposed. The algorithm was developed with reference to the charge-flipping method (Oszlányi & Sütő, 2008 ▸; Palatinus, 2013 ▸). It is emphasized that the iterative algorithm opens the possibility of the application to structures without centrosymmetry, which tremendously widens the search space of phase combinations. The method was tested for LLC bicontinuous cubic phases and mesoporous silicas, and structures were successfully determined for all tested data, although additional constraints were necessary in some cases. Notably, the method converged to the proper structures without information on the space group.

## 2. Phase retrieval method

The algorithm was designed to find the structure or structure-factor phase with the smallest difference between the maximum and minimum electron densities in a unit cell. The electron density was calculated in a unit cell of 32 × 32 × 32 voxels. All calculations were performed using a home-made script in python3.

A finite number of structure-factor amplitudes 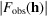 are observed in crystal diffraction experiments. Unobserved amplitudes, including 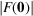 , were set to zero in the iterative process. 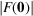 cannot be determined in principle because no zero-electron-density region is observed in the target sample. Since 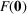 is not included, the electron density, 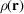 , in the unit cell has positive and negative values and its mean is 0.

In the iterative calculations, the initial phases were given random values in the range of −π to π which satisfy the Friedel law. When the space group was used as a constraint on the initial phases, the phases were assumed to be random to the extent that these satisfy the phase relations in reciprocal space (Shmueli *et al.*, 2010 ▸). The initial structure factor is as follows:

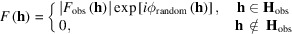

where** H**_obs_ is the set of reciprocal-lattice vectors **h** for which the structure factor was observed.

The following calculation steps from (i) to (iv) are repeated:

(i) 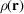 is calculated by determining the Fourier transform of 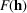 ,

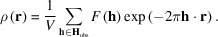




(ii) 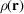 is modified as follows to obtain 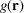 :

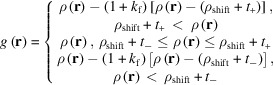

where *k*_f_ is the flipping parameter and 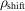 is the magnitude of the electron-density shift. When the volume fraction of the positive region *v*_p_ is not set, 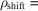 0. *t*_+_ and *t*_−_ are the upper and lower thresholds. 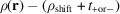 in the equation is the amount above or below the threshold. The flipping parameter *k*_f_ is often set between 0 and 1. When *k*_f_ = 0, the density outside the upper and lower thresholds is replaced by the threshold value: 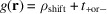 . When *k*_f_ = 1, it is replaced by the threshold minus the amount above or below the threshold: 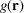 = 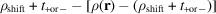 = 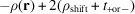 . The upper and lower threshold values are 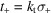 and 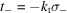 . When 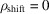 , 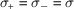 , where σ is the standard deviation of 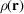 . When 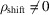 , 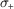 is the root-mean-square of 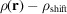 in the region 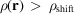 , and 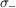 is the root-mean-square of 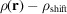 in the region 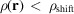 . 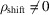 only when *v*_p_ is set. *v*_p_ is the volume fraction in the unit cell where 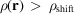 and takes values between 0 and 1. The threshold parameter *k*_t_ is often set from 0.2 to 1.3.

(iii) 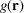 is Fourier transformed to obtain the structure factor, 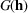 = 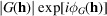 .

(iv) A new structure factor is obtained as follows:

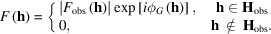




When *k*_t_ = 1 and 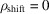 , the upper and lower threshold values *t*_+_ and *t*_−_ are σ, the standard deviation of 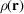 . Thus, most final structures have electron densities outside the upper and lower thresholds. When the parameters *k*_f_ and *k*_p_ were fixed in the calculation, the rate of obtaining the correct solution was low. Therefore, during the calculation cycle, they were changed. In the *j*th cycle, the parameters were as follows: 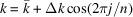 , where 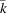 , 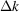 and *n* are the mean, width and period of a parameter *k*, respectively. Different values were set for *n*_f_ and *n*_p_ so that the periods of parameter changes of *k*_f_ and *k*_p_ do not coincide.

If the structure is known to have centrosymmetry, it is possible to add the constraint that the structure factor be real. In this case, the structure factor in step (iv) becomes

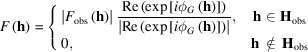

where Re() is a function that extracts the real part of the value in the parentheses. The progress of the iterative calculation can be monitored by the difference between the maximum and minimum electron densities in the unit cell, 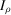 = 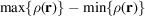 (Oka, 2022 ▸). Calculation steps are repeated a set number of iterations, and the final structure is the one with the minimum 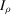 during the calculation. Multiple independent calculations may produce different results. Also, the origin-shifted structures are outputted even if the structures are completely equivalent.

The output results were evaluated using 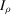 and 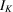 . 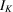 , an indicator based on the convexity of the electron density, is described in detail in the previous paper (Oka, 2022 ▸) and briefly here. In materials with TPMS-like structures, convex regions, *i.e.* regions with closed isoelectron-density surfaces, are considered to be small. The convex regions can be determined by the eigenvalues of the Hessian matrix of the electron density, 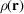 :

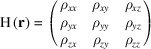

where the subscripts indicate partial derivatives. If the signs of the eigenvalues of the Hessian matrix are all the same, then the region is strictly convex (Rockafellar & Wets, 2010 ▸). Let *C* be the electron-density regions that are strictly convex. Then, the indicator *I_K_* is defined by 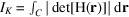 . Both indicators have been shown to be useful in the structure determination of LLC bicontinuous cubic phases (Oka, 2022 ▸). When both of these indicators are small and close values are obtained in several independent calculations, it can be presumed that the proper structure is found.

The phases of the outputted structure can be compared with that of the true structure by the following *R*_p_ value (Oka, 2022 ▸):

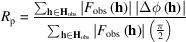

where 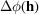 = 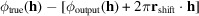 [ 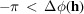 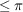 ]. The origin shift **r**_shift_ of the outputted structure was obtained by minimization of 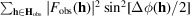 . *R*_p_ approaches 0 as the phase agreement becomes higher. According to Babinet’s principle, when the electron density of a sample is inverted, the diffraction intensity is equivalent to that before the inversion. For this reason, a structure and a density-inversed structure are equivalent, and the smaller *R*_p_ of the two structures is adopted as the *R*_p_ of the structure. Thus, *R*_p_ is a value between 0 and 1.

## 3. Structure determination examples

### 3.1. Phase retrieval with a centrosymmetric space-group constraint

Table 1 ▸ lists the 11 data sets used in the structure determination. The six LLC bicontinuous cubic phases correspond to X-ray diffraction data from single crystals we have measured previously (Oka, 2017 ▸; Oka *et al.*, 2018 ▸, 2020 ▸). These are considered to be accurate with regard to the phase of the structure factors, which is obtained by optimizing the model to the X-ray diffraction data. As examples other than the LLC bicontinuous cubic phases, the structures of mesoporous silicas were determined. Four of the mesoporous silica data sets were obtained using high-resolution transmission electron microscopy (Sakamoto *et al.*, 2004 ▸; Gao *et al.*, 2006 ▸; Zhang *et al.*, 2011 ▸; Cao *et al.*, 2016 ▸), and the structure factors, including phase, are considered reliable. The data for MCM-48 were obtained by powder X-ray diffraction at a synchrotron radiation facility, and the phase of the structure factor was obtained by model optimization (Solovyov *et al.*, 2005 ▸). Therefore, the phases are reliable.

Materials with TPMS-like structures generally have large structural disorder, and the spatial resolution of the data obtained is lower than for solid crystals. Also, due to their high symmetry, the number of independent reflections is not large (Table 1 ▸). In the type-I LLC bicontinuous cubic phase, polar regions with high electron density gather on the TPMS and non-polar regions with low electron density gather on the network side; in type II, the positions of polar and non-polar regions are opposite to those in type I (Hyde *et al.*, 1996 ▸). In all mesoporous silicas in Table 1 ▸, with one exception, the silica walls are located on the TPMS and the network sides are vacant. Only PEO_117_-b-PS_77_-PtBA_179_ templated mesoporous silica has a single-gyroid structure with silica located on only one of the two **srs** nets, the other being vacant (Cao *et al.*, 2016 ▸). Therefore, this structure is chiral and not centrosymmetric, whereas all the other structures are centrosymmetric. The volume fraction of the TPMS side of each sample is shown in Table 1 ▸.

First, the structural determination was tried using the constraint of centrosymmetry and using as few other constraints as possible. The initial phases were set to random values within the range satisfying the phase relationship in each centrosymmetric space group (Shmueli *et al.*, 2010 ▸). Since the space groups are centrosymmetric, the constraint that the structure factor be real in the iterative processes was used. A structure lacking centrosymmetry was not tried here because of the complexity of the initial constraints derived from the space group. The results of the structure determinations are summarized in Table 2 ▸. For eight data sets, out of 100 independent calculations without additional constraints, structures with sufficiently small *R*_p_ were obtained for all of them. The *R*_p_ were calculated between the obtained structures and the previously determined true structures.

**Figure 1:**
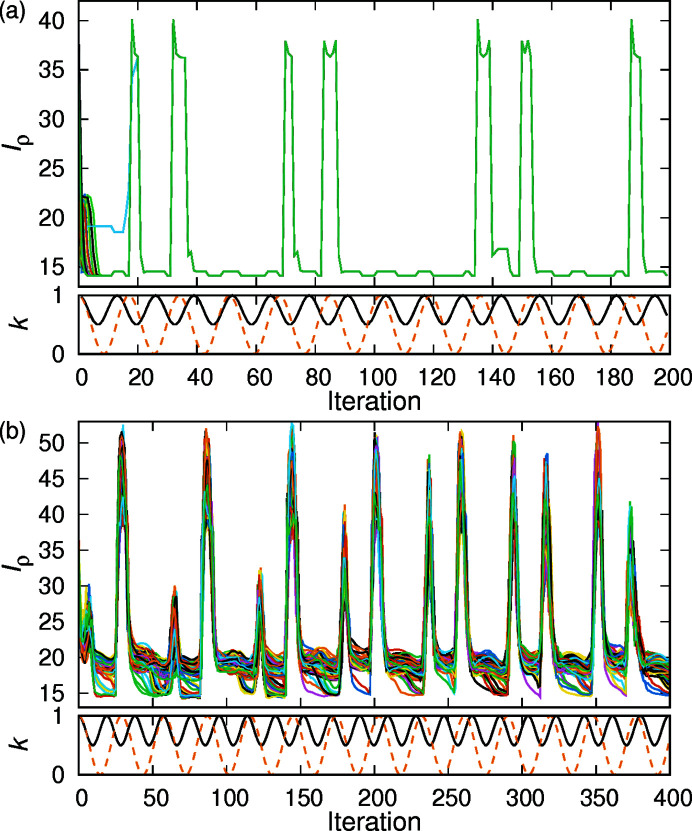
Changes of the indicator *I*_ρ_ and the parameters *k*_t_ and *k*_f_ during the calculation of structure determination in phytantriol 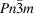 . Parameter settings are listed in Table 2 ▸ (*a*) and Table 3 ▸ (*b*). The tops of the figures show the changes in *I*_ρ_ for 100 independent calculations in various colors. At the bottom of the figures, the changes in *k*_t_ are shown as a solid black line and *k*_f_ as a dashed orange line.

Fig. 1 ▸(*a*) shows the changes in the indicator *I*_ρ_ and the parameters *k*_t_ and *k*_f_ during the iterative calculation process of structure determination in the data of phytantriol 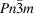 . *I*_ρ_ was useful as an indicator to show the progress of structure determination, since *I*_ρ_ appeared to be minimized in the iterative process. The top part of the figure shows the change in *I*_ρ_ over 100 independent calculations. The *I*_ρ_ values for those 100 independent calculations converge to two value traces when the number of iterations is less than 10, and to a single trace at about 20 iterations. Thereafter, the value of *I*_ρ_ may increase temporarily in response to changes in the parameters *k*_f_ and *k*_t_, but it remains close to the lowest value in many regions. Although the value of *I*_ρ_ fluctuated during the iterative calculations, the structure with minimum *I*_ρ_ was adopted as the final solution. The lower part of the figure shows changes in the parameters *k*_f_ and *k*_t_, which were changed periodically in the iterative process. The reason for the periodic changes of both parameters was to perform calculations with different combinations of parameters and to avoid staying in local minima. Fixing *k*_f_ and *k*_t_ in the iterative process often resulted in convergence to several different local minima, which frequently did not result in the proper structure.

**Figure 2:**
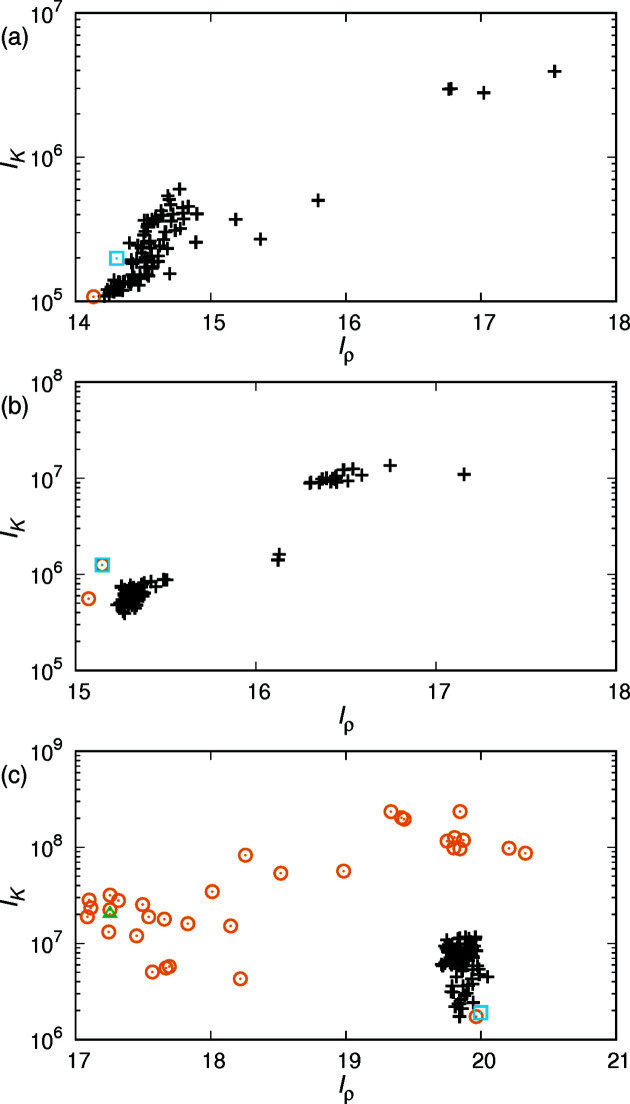
The scatter plot of *I*_ρ_ and *I_K_* for the structures obtained from 100 independent calculations in phytantriol 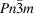 (*a*) and 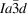 (*b*) and C_12_EO_6_ (*c*). The orange circles were obtained with the parameters in Table 2 ▸, and the black crosses were obtained with those in Table 3 ▸. The sky-blue squares are the values of the true structures obtained previously. The green triangle in (*c*) was obtained with the parameters in Table 2 ▸ other than *v*_p_.

The *I*_ρ_ and *I_K_* values for the structures obtained from the phytantriol 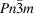 and 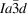 data are shown in Figs. 2 ▸(*a*) and 2 ▸(*b*). The true and obtained structures have very close or coincident *I*_ρ_ and *I_K_* values. The previous study showed that the *I*_ρ_ and *I_K_* values of the true structure have values close to the minimum (Oka, 2022 ▸). The structure obtained in this study [Fig. 2 ▸(*a*)] is consistent with the structure with the minimum values of both *I*_ρ_ and *I_K_* previously obtained for phytantriol 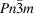 . The two obtained structures in phytantriol 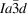 [Fig. 2 ▸(*b*)] also agree with the first and second minimum structures previously obtained.

Each data set for monoolein in three space groups showed a single final structure (Table 2 ▸). For 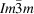 and 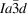 , the *R*_p_ value was 0, which is in perfect agreement with the true structure. On the other hand, 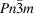 had *R*_p_ = 0.080, the highest minimum *R*_p_ among the data used in this study. This is probably due to the fact that the true structure in 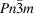 is *I_K_*-minimal but not *I*_ρ_-minimal, as shown in a previous paper (Oka, 2022 ▸). The mesoporous silica, except for MCM-48, yielded a final solution close to the true structure (Table 2 ▸).

For C_12_EO_6_ and MCM-48, the proper structure could not be obtained without additional constraints. The proper structure was obtained when the parameter *v*_p_ was set. When *v*_p_ = 0.75 was used in C_12_EO_6_, structures close to the true structure were obtained 19 times out of 100 independent calculations. Fig. 2 ▸(*c*) shows the distribution of *I*_ρ_ and *I_K_* for the 100 structures obtained from the C_12_EO_6_ calculation; the 19 indicator points [overlapped by a single point in Fig. 2 ▸(*c*)] almost overlap with the point of the true structure. However, the *I*_ρ_ and *I_K_* of the other points are widely distributed, but many of them have smaller *I*_ρ_ than the *I*_ρ_ of the true structure. Many points are also distributed around the indicator values of the structure determined without using the *v*_p_ constraint. This result is consistent with a previous study showing that the *I*_ρ_ of the true structure is not minimal in C_12_EO_6_. Therefore, *I*_ρ_ cannot be used as an indicator of the true structure in C_12_EO_6_. On the other hand, as shown in previous studies, *I_K_* is valid as an indicator, and the structure with the minimum *I_K_* among the obtained structures is close to the true structure. The true structure was always obtained when *v*_p_ = 0.25 in MCM-48. The *I*_ρ_ of the obtained structure was larger than that when *v*_p_ was not set, but *I_K_* was smaller (data not shown). It has been previously shown that when the volume fraction of the TPMS side is above 0.7, *I*_ρ_ is less valid as an indicator of structure and *I_K_* is superior to *I*_ρ_ (Oka, 2022 ▸). These examples suggest that if the volume fraction of the high (or low) electron-density region is far from 0.5, it is better to set an additional constraint *v*_p_. It is also better to focus on *I_K_* rather than *I*_ρ_ in determining the final structure when *v*_p_ needs to be set. A reminder about *v*_p_ here: *v*_p_ determines the zero position of the electron density when calculating the standard deviation, and is one of the parameters similar to *k*_f_ and *k*_t_. It is recommended to set a value close to the real volume fraction, but it is not necessary to set it to the same value. It would be better to adjust *v*_p_ based on the results of iterative calculations.

### 3.2. Phase retrieval without a space-group constraint

Next, the structure was determined without using the space-group constraint and with as few other constraints as possible. The results are summarized in Table 3 ▸. Under the condition of no constraints, proper structures with *R*_p_ < 0.1 were obtained with more than half probability for six data sets. To obtain the proper structures in the other data, constraints were necessary.

An example of data for which a proper structure was obtained under completely unconstrained conditions is shown for phytantriol 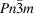 . The change in *I*_ρ_ during the iterative process is shown in Fig. 1 ▸(*b*). The value traces of *I*_ρ_ repeatedly increase and decrease, but some of them reach a minimum value around *I*_ρ_ = 14 after a large increase in *I*_ρ_. Calculations with fixed parameters *k*_f_ and *k*_p_ were also tried: *I*_ρ_ was often trapped at a certain value and *I*_ρ_ did not decrease further even if the number of calculations was increased. On the other hand, when *k*_f_ and *k*_p_ were changed periodically, the search for a solution was efficient. In addition, the calculation time was acceptable for the current condition. Therefore, the parameters *k*_f_ and *k*_p_ were changed periodically. The data of phytantriol 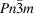 yielded 56 structures with *R*_p_ < 0.1 in 100 independent calculations over 700 iterations (Table 3 ▸). Scatter plots of *I*_ρ_ and *I_K_* are shown in Fig. 2 ▸(*a*). The values of the obtained structures are distributed around that of the true structure. The distribution of the obtained values appears to be in the process of converging to the value of the structure using the space group as a constraint. When the number of iterations was set to 2800, the number of structures with *R*_p_ < 0.1 increased to 92 out of 100 calculations. If the number of iterations is set to a larger value, the distribution will probably converge to one or a few points. For phytantriol 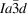 , 80 of 100 independent calculations resulted in *R*_p_ < 0.1, with a minimum *R*_p_ of 0.021 (Table 3 ▸). The scatter plot, Fig. 2 ▸(*b*), shows that the *I*_ρ_ and *I_K_* distributions for the final structure are concentrated near the true structure.

For the monoolein 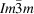 and 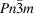 data, the structure factor had to be constrained to a real number because without the constraint the proper structure could not be obtained. Compared with the other data, the result of monoolein may have been affected by the small number of independent reflections. On the other hand, monoolein 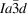 , with the smallest number of independent reflections, converged to a nearly true structure without constraints. The ease of convergence may differ for each space group.

For the volume fraction of the LLC bicontinuous cubic phase, C_12_EO_6_ has the largest value of 0.72 (Table 1 ▸). A volume fraction constraint was necessary for C_12_EO_6_. When calculated with *v*_p_ = 0.75, the distribution of *I*_ρ_ and *I_K_* for the obtained structure is shown in Fig. 2 ▸(*c*). The values of the obtained structures are distributed around that of the true structure. Also, under this condition, all calculations yielded structures with *R*_p_ < 0.1. In the calculation with *v*_p_ = 0.72, the structure with *R*_p_ < 0.1 was obtained in 60 out of 100 independent calculations (Table 3 ▸). Better results were obtained for C_12_EO_6_ by changing *v*_p_ slightly from the actual volume fraction.

The three mesoporous silicas, AMS-10, EO_20_PO_70_EO_20_ templated and IBN-9, yielded final structures close to the true structure without constraints. The volume fractions of silica in these structures ranged from 0.283 to 0.412, relatively close to 0.5 compared with the value of 0.25 for MCM-48 (Table 1 ▸). MCM-48 required two constraints: a real number for the structure factor and a set volume fraction. When the number of iterations was 700, only five out of 100 independent calculations yielded a structure with *R*_p_ < 0.1. Then, when the number of iterations was set to 7000, the number increased to 72 (Table 3 ▸). It seems that as iterations are increased, the number of final structures that are close to the true structure increases. For PEO_117_-b-PS_77_-PtBA_179_ templated, 100 independent calculations yielded 96 structures with *R*_p_ < 0.1. Since the structure is chiral, this value includes the mirror image structure; excluding the mirror image, the value is 45. This calculation required the constraint of *v*_p_; the best *I_K_* values for the final structures were obtained when *v*_p_ = 0.40. The reason for the necessity of the *v*_p_ constraint is probably due to the large number of independent reflections.

**Figure 3:**
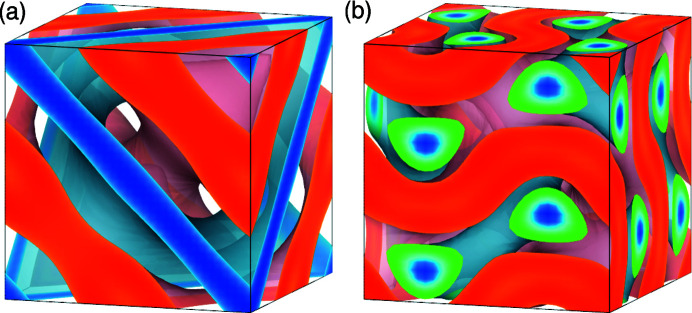
Electron densities of (*a*) phytantriol/water ( 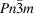 ) and (*b*) C_12_EO_6_ with the minimum *I*_*K*_ among those calculated under the conditions in Table 3 ▸. They are translationally shifted so that the origins of the unit cells are the same as those of the expected space groups. Isodensity surfaces are drawn with a volume fraction of (*a*) 0.25 and (*b*) 0.55 on high-density sides (pale red) and 0.25 on low-density sides (pale blue). In the cross section, the highest electron-density regions are depicted in red and the lowest in blue. Electron densities were drawn using *VESTA* (Momma & Izumi, 2011 ▸).

The obtained electron densities of phytantriol/water ( 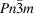 ) and C_12_EO_6_ are shown in Fig. 3 ▸. The electron densities are the translationally shifted ones obtained by phase retrieval. These are in good agreement with the previously determined electron densities (Figs. S1 and S2 in the supporting information), although the space group was not used.

Without the use of space groups, nearly true structures were obtained without any constraints in more than half of the cases. Even when iteration did not work without constraints, the constraints of a real structure factor or set *v*_p_ yielded a nearly true structure. Judging from this result, this structure determination method can be used for a TPMS-like structure even when the space group is not known.

In this paper, the electron density was calculated in a unit cell of 32 × 32 × 32 voxels. The minimum grid spacing required for the charge-flipping method is *d*_min_/2 (Oszlányi & Sütő, 2004 ▸), where *d*_min_ is the minimum interplanar distance. Thus, the minimum required number of divisions per one edge of the unit cell is *d*_min_/2*a*, where *a* is a lattice constant. In the data used in this paper, the minimum required number is 10–22. Since a small number of divisions results in a poor representation of the electron density and affects the values of the indicators *I*_ρ_ and *I*_*K*_, 32 divisions were used.

## 4. Conclusion

An algorithm for determination of TPMS-like structures from diffraction data was developed and shown to be effective. Although additional constraints were necessary in some cases, structure determination was achieved for all tested data sets. The obtained structures were close to the true structure, and *R*_p_, which indicates phase difference, was close to zero. The algorithm appears to efficiently find the structure with the smallest *I*_ρ_ by cutting or inverting the electron density outside the upper and lower thresholds. Constraints on *v*_p_ were necessary when the volume fraction was far from 0.5 and when the number of independent reflections was large. For some parameter settings, several different structures were obtained, but by using *I_K_* in conjunction with *I*_ρ_, the proper structure could be determined. Previous studies have shown that *I_K_* is superior to *I*_ρ_ in determining TPMS-like structures. Therefore, when multiple structure candidates are obtained, *I_K_* should also be used as a reference.

The determination was more efficient when information on centrosymmetric space groups was used. Therefore, when using this algorithm to determine unresolved structures, it appears better to use known information on space groups. On the other hand, this is not essential in structure determination. Thus, it is possible to determine a structure for which the space group is unknown. The number of independent reflections in the data sets for the space group 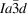 was 8 for monoolein and 39 for MCM-48, so the relative spatial resolution was low for monoolein and high for MCM-48. Thus, the method appears to be applicable independently of spatial resolution and the number of independent reflections. The previously reported method requires a long computation time when the number of independent reflections is large, so the iterative method reported here should be used in such cases.

Although the LLC bicontinuous cubic phase and mesoporous silica were used here, this method could be applied to TPMS-like structures such as thermotropic liquid crystals and polymers. If there exists a three-dimensional periodic structure with bicontinuous or polycontinuous regions different from the TPMS-like structure, the method may be applicable to such a system as well.

## Supplementary Material

Supporting information. DOI: 10.1107/S2053273322010786/ik5006sup1.pdf

## Figures and Tables

**Table 1:** Data used in structure determination In the last column (volume fraction), the value is the volume fraction of the low-electron-density region in the LLC type II, while it is the volume fraction of the high region in the other samples.

Classification	Sample	TPMS	Space group	Lattice constant (nm)	Min. interplanar distance (nm)	Independent reflections	Volume fraction (TPMS side)
LLC type II	Monoolein/water (Oka, 2017 ▸)	P	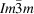 †	14.19	2.90	12	0.43
		D	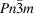 †	11.26	2.40	14	0.44
		G	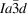 †	14.65	2.87	8	0.54
	Phytantriol/water (Oka *et al.*, 2018 ▸)	D	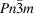 †	6.474	1.202	21	0.57
		G	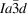 †	8.748	1.169	21	0.66
LLC type I	C_12_EO_6_/water (Oka *et al.*, 2020 ▸)	G	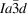 †	11.32	1.512	21	0.72
Mesoporous silica	AMS-10 (Gao *et al.*, 2006 ▸)	D	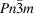 †	9.6	1.35	18	0.412
	EO_20_PO_70_EO_20_ templated (Sakamoto *et al.*, 2004 ▸)	G	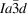 †	23.8	2.62	25	0.283
	MCM-48 (A-SY) (Solovyov *et al.*, 2005 ▸)	G	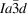 †	9.661	1.030	39	0.25
	IBN-9 (Zhang *et al.*, 2011 ▸)	H	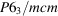 †	*a* = *b* = 8.84, *c* = 8.43	1.91	12	0.387
	PEO_117_-b-PS_77_-PtBA_179_ templated (Cao *et al.*, 2016 ▸)	G	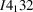	70	6.2	50	0.3‡

† Centrosymmetric space groups.

‡ Volume fraction of silica on one gyroid network region. The other gyroid network region (including TPMS) is void.

**Table 2:** Structure determination results using centrosymmetric space groups as initial random phase constraints Structure factors are restricted to real numbers during the calculation in all structure determinations. The penultimate column gives the number of structures out of the 100 obtained that satisfy *R*_p_ < 0.1.

Sample	Space group	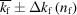	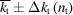	Iterations	Additional constraint	*R*_p_ < 0.1	Min. *R*_p_
Monoolein/water	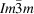	0.25±0.25 (17)	0.6±0.4 (13)	200	–	100†	0
	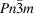	0.75±0.25 (17)	0.6±0.4 (13)	200	–	100†	0.080
	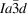	0.75±0.25 (17)	0.75±0.25 (13)	200	–	100†	0
Phytantriol/water	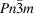	0.75±0.25 (17)	0.75±0.25 (13)	200	–	100†	0.015
	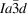	0.75±0.25 (17)	0.75±0.25 (13)	200	–	100	0
C_12_EO_6_/water	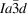	0.25±0.25 (17)	0.75±0.25 (13)	200	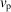 = 0.75	19	0.005
AMS-10	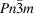	0.75±0.25 (17)	0.75±0.25 (13)	200	–	100†	0.003
EO_20_PO_70_EO_20_ templated	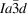	0.75±0.25 (17)	0.75±0.25 (13)	200	–	100	0.018
MCM-48	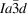	0.75±0.25 (29)	0.6±0.4 (19)	700	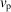 = 0.25	100	0
IBN-9	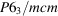	0.75±0.25 (17)	0.75±0.25 (13)	200	–	100†	0.056

† Only one solution was obtained.

**Table 3:** Structure determination results without using the space group as a constraint on the initial phase The penultimate column gives the number of structures out of the 100 obtained that satisfy *R*_p_ < 0.1.

Sample	Space group	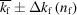	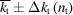	Iterations	Additional constraint	*R*_p_ < 0.1	Min. *R*_p_
Monoolein/water	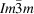	0.5±0.5 (29)	0.75±0.25 (19)	700	Real *F* †	96	0
	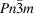	0.5±0.5 (29)	0.6±0.4 (19)	700	Real *F* †	60	0.065
	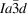	0.5±0.5 (29)	0.75±0.25 (19)	700	–	76	0.001
Phytantriol/water	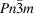	0.5±0.5 (29)	0.75±0.25 (19)	700	–	56	0.042
	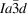	0.5±0.5 (29)	0.75±0.25 (19)	700	–	80	0.021
C_12_EO_6_/water	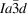	0.5±0.5 (17)	0.75±0.25 (13)	400	*v*_p_ = 0.75	100	0.041
AMS-10	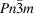	0.5±0.5 (29)	0.6±0.4 (19)	700	–	92	0.002
EO_20_PO_70_EO_20_ templated	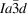	0.5±0.5 (29)	0.75±0.25 (19)	700	–	92	0.020
MCM-48	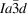	0.5±0.5 (29)	0.65±0.35 (19)	7000	Real *F* †, *v*_p_ = 0.25	72	0
IBN-9	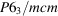	0.5±0.5 (29)	0.75±0.25 (19)	700	–	56	0.060
PEO_117_-b-PS_77_-PtBA_179_ templated	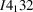	0.5±0.5 (29)	0.65±0.35 (19)	7000	*v*_p_ = 0.40	96‡	0.050

† Structure factors are restricted to real numbers during the calculation.

‡ Sum of two chiral (mirror image) structures.
