# Supplementary material for: A phase retrieval algorithm for triply periodic minimal surface like structures
Source: Acta Crystallogr A Found Adv. 2023 Jan 1;79(Pt 1):51–8. doi: 10.1107/S2053273322010786 (PMC9813685; doi:10.1107/S2053273322010786)
Supplement: Supplementary file 1 [file a-79-00051-sup1.pdf]

Acta Cryst A
FOUNDATIONS
ADVANCES

Volume 79 (2023)

Supporting information for article:

A phase retrieval algorithm for triply periodic minimal surface like structures

Toshihiko Oka

Contents

Figure S1. Electron densities of phytantriol/water ($Pn\bar{3}m$).

Figure S1. Electron densities of $C_{12}EO_6$ ($Ia\bar{3}d$).

Table S1. Phases phytantriol/water ($Pn\bar{3}m$) obtained by the phase retrieval method.

Table S2. Phases of $C_{12}EO_6$ ($Ia\bar{3}d$) obtained by the phase retrieval method.

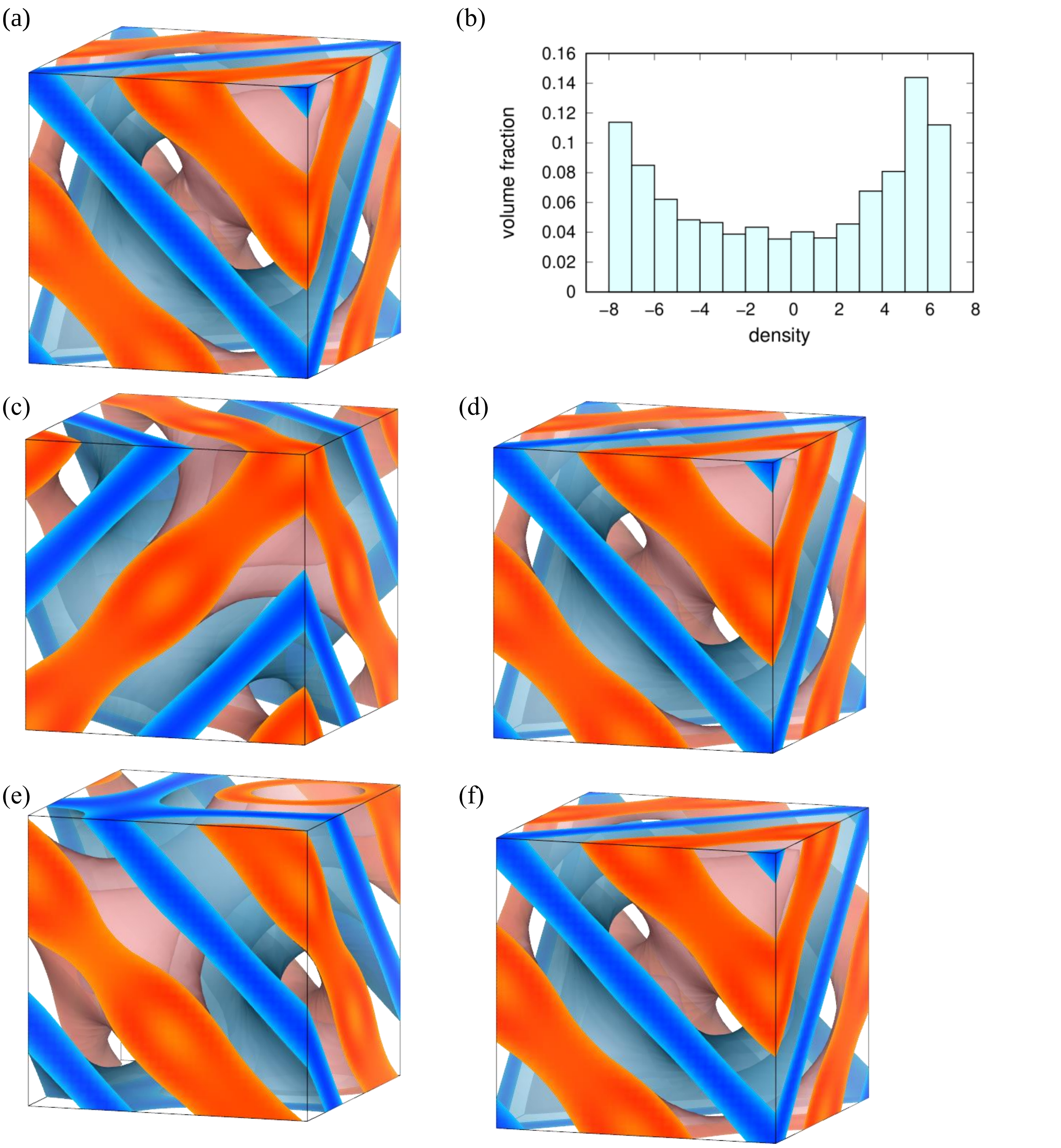

Figure S1. Electron densities of phytantriol/water ($Pn\bar{3}m$), (a)data taken from oka et al.[1], (b)histogram of (a), (c)I_K minimum solution with centrosymmetric space group constraint, (d)translational shifted (c), (e) I_K minimum solution without constraint, and (f) translational shifted (e). Isodensity surfaces are drawn with a volume fraction of 0.25 (ρ=5.1) on high density sides (pale red) and 0.25 (ρ=-5.2) for on density sides (pale blue). In the cross-section, the highest electron density regions are depicted in red and the lowest in blue. Electron densities were created using VESTA[2].

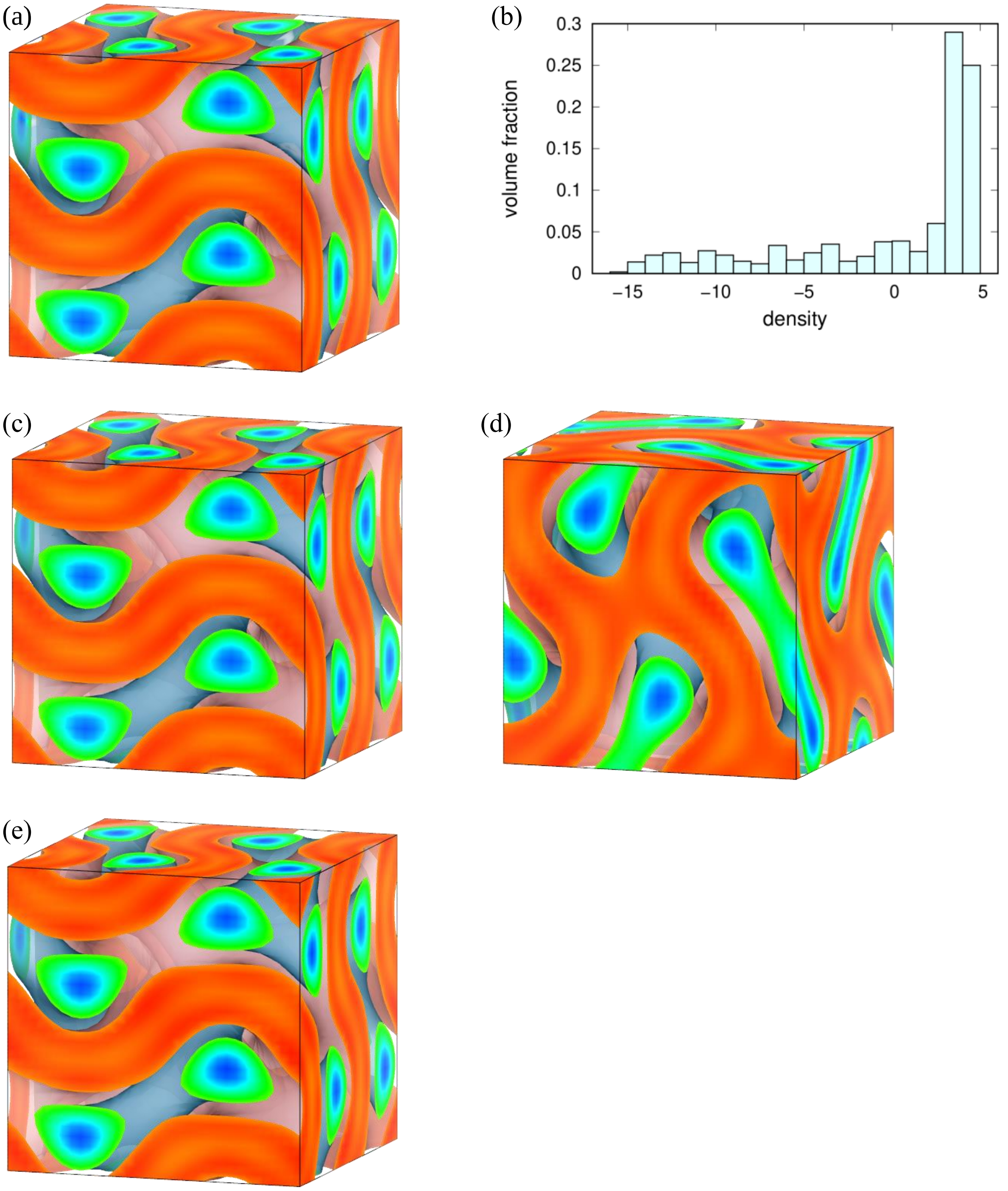

Figure S2. Electron densities of $C_{12}EO_6$ ($Ia\bar{3}d$), (a)data taken from oka et al.[3], (b)histogram of (a), (c)I_K minimum solution with centrosymmetric space group constraint, (d)I_K minimum solution without constraint, and (e) translational shifted (d). Isodensity surfaces are drawn with a volume fraction of 0.55 (ρ=2.8) on high density sides (pale red) and 0.25 (ρ=-3.1) on low density sides (pale blue). In the cross-section, the highest electron density regions are depicted in red and the lowest in blue. Electron densities were created using VESTA[2].

Table S1. Phases phytantriol/water ($Pn\bar{3}m$) obtained by the phase retrieval method.

The position of the obtained structure (minimum I_K) by the phase retrieval method were aligned to the reference structure, and the mean and standard deviation of the phases are calculated using the phase relation of the space group[4].

sample				phytantriol/water					
space group				$Pn\bar{3}m$ (224)					
				data taken from oka et al.[1]		centrosymmetric space group constraint*		no constraint[#]	
h	k	l	m	$\|F_{obs}\|$	ϕ/π	$\bar{\phi}/\pi$	$\sigma_\phi{}^\dagger/\pi$	$\bar{\phi}/\pi$	$\sigma_\phi{}^\dagger/\pi$
1	1	0	12	1.000	1	1	0	-0.998	0.002
1	1	1	8	0.953	1	1	0	0.993	0.004
2	0	0	6	0.517	0	0	0	0.007	0.015
2	1	1	24	0.3094	1	1	0	0.99	0.03
2	2	0	12	0.250	1	1	0	-0.987	0.010
2	2	1	24	0.2233	1	1	0	0.991	0.015
3	1	0	24	0.0741	0	0	0	0.00	0.03
3	1	1	24	0.02192	0	0	0	-0.010	0.010
2	2	2	8	0.0998	1	1	0	0.962	0.02
3	2	1	48	0.01237	1	1	0	1.00	0.19
4	0	0	6	0.0110	1	0	0	0.05	0.12
3	2	2	24	0.01113	1	0	0	0.04	0.09
3	3	0	12	0.0171	0	0	0	-0.04	0.03
4	1	1	24	0.01248	1	1	0	-0.98	0.05
3	3	1	24	0.02027	0	0	0	-0.026	0.018
4	2	0	24	0.01448	1	1	0	1.00	0.03
4	2	1	48	0.01045	1	1	0	1.00	0.03
3	3	2	24	0.0268	0	0	0	-0.014	0.008
4	3	1	48	0.0054	0	0	0	0.00	0.02
3	3	3	8	0.0216	0	0	0	-0.018	0.010
4	3	2	48	0.00809	0	0	0	0.00	0.02
R_p					-	0.015		0.043	

* See Table 2 for details of conditions. # See Table 3 for details of conditions. †Standard deviation.

Table S2. Phases of $C_{12}EO_6$ ($Ia\bar{3}d$) obtained by the phase retrieval method.

The position of the obtained structure (minimum I_K) by the phase retrieval method were aligned to the reference structure, and the mean and standard deviation of the phases are calculated using the phase relation of the space group[4].

sample				$C_{12}EO_6$					
space group				$Ia\bar{3}d$ (230)					
				data taken from oka et al.[3]		centrosymmetric space group constraint*		no constraint[#]	
h	k	l	m	$\|F_{obs}\|$	ϕ/π	$\bar{\phi}/\pi$	$\sigma_\phi^\dagger/\pi$	$\bar{\phi}/\pi$	$\sigma_\phi^\dagger/\pi$
2	1	1	24	1.000	0	0	0	0.000	0.005
2	2	0	12	0.555	0	0	0	0.000	0.008
3	2	1	48	0.0371	0	0	0	0.0	0.4
4	0	0	6	0.1781	0	0	0	-0.02	0.06
4	2	0	24	0.1813	0	0	0	0.001	0.016
3	3	2	24	0.2454	1	1	0	-0.998	0.009
4	2	2	24	0.1477	1	1	0	0.999	0.010
4	3	1	48	0.0958	1	1	0	1.000	0.014
5	2	1	48	0.0214	0	0	0	0.00	0.03
4	4	0	12	0.0144	0	0	0	0.01	0.07
5	3	2	48	0.0195	0	0	0	0.00	0.019
6	1	1	24	0.0359	0	0	0	0.000	0.014
6	2	0	24	0.0061	0	1	0	-0.01	0.06
5	4	1	48	0.0207	0	0	0	0.000	0.011
6	3	1	48	0.0189	0	0	0	0.00	0.03
4	4	4	8	0.0412	1	1	0	1.000	0.008
5	4	3	48	0.0267	1	1	0	1.00	0.02
6	4	0	24	0.0122	0	0	0	-0.01	0.07
5	5	2	24	0.0117	1	1	0	1.00	0.09
6	3	3	24	0.0081	1	1	0	1.00	0.12
6	4	2	48	0.0057	1	1	0	1.00	0.14
R_p					-	0.005		0.041	

* See Table 2 for details of conditions. # See Table 3 for details of conditions. †Standard deviation.

Reference

(1) Oka, T.; Ohta, N.; Hyde, S. Polar–Nonpolar Interfaces of Inverse Bicontinuous Cubic Phases in Phytantriol/Water System Are Parallel to Triply Periodic Minimal Surfaces. *Langmuir* **2018**, *34* (50), 15462–15469. https://doi.org/10.1021/acs.langmuir.8b03320.

(2) Momma, K.; Izumi, F. VESTA 3 for Three-Dimensional Visualization of Crystal, Volumetric and Morphology Data. *J. Appl. Crystallogr.* **2011**, *44* (6), 1272–1276. https://doi.org/10.1107/S0021889811038970.

(3) Oka, T.; Ohta, N.; Hyde, S. T. Polar–Nonpolar Interfaces of Normal Bicontinuous Cubic Phases in Nonionic Surfactant/Water Systems Are Parallel to the Gyroid Surface. *Langmuir* **2020**, *36* (30), 8687–8694. https://doi.org/10.1021/acs.langmuir.0c00597.

(4) Shmueli, U.; Hall, S. R.; Grosse-Kunstleve, R. W. Symmetry in Reciprocal Space. In *International Tables for Crystallography Volume B: Reciprocal space*; 2010; pp 114–174.
